# The association of antidiabetic medications and Mini-Mental State Examination scores in patients with diabetes and dementia

**DOI:** 10.1186/s13195-021-00934-0

**Published:** 2021-12-02

**Authors:** Juraj Secnik, Hong Xu, Emilia Schwertner, Niklas Hammar, Michael Alvarsson, Bengt Winblad, Maria Eriksdotter, Sara Garcia-Ptacek, Dorota Religa

**Affiliations:** 1 Division of Clinical Geriatrics, Center for Alzheimer Research, Department of Neurobiology, Care Sciences and Society, Karolinska Institutet, Neo, Blickagången 16, 14152 Huddinge, Sweden; 2 Department of Neurology, Second Faculty of Medicine, Charles University, Motol University Hospital, Prague, Czech Republic; 3 Department of Medical Epidemiology and Biostatistics, Karolinska Institutet, Stockholm, Sweden; 4 Institute of Environmental Medicine, Karolinska Institutet, Stockholm, Sweden; 5 Growth and Metabolism, Department of Molecular Medicine and Surgery, Karolinska Institutet, Stockholm, Sweden; 6 Division of Neurogeriatrics, Center for Alzheimer Research, Department of Neurobiology, Care Sciences and Society, Karolinska Institutet, Stockholm, Sweden; 7 Theme Aging, Karolinska University Hospital, Huddinge, Sweden

**Keywords:** Diabetes, Dementia, Antidiabetics, Metformin, DPP-4i, MMSE

## Abstract

**Background:**

The effect of antidiabetic medication on cognitive function is unclear. We analyzed the association between five antidiabetic drugs and change in Mini-Mental State Examination (MMSE) scores in patients with diabetes and dementia.

**Methods:**

Using the Swedish Dementia Registry and four supplementary Swedish registers/databases, we identified 1873 patients (4732 observations) with diagnosis of type 2 diabetes (diabetes) and Alzheimer’s disease or mixed-pathology dementia who were followed up at least once after dementia diagnosis. Use of metformin, insulin, sulfonylurea, thiazolidinediones (TZD), and dipeptidyl-peptidase-4 inhibitors (DPP-4i) was identified at baseline. Prevalent-user, incident-user, and drug-drug cohorts were sampled, and propensity-score matching was used to analyze comparable subjects. Beta coefficients with 95% confidence intervals (*CI*) from the random intercept and slope linear mixed-effects models determined the association between the use of antidiabetic medications and decline in MMSE score points between the follow-ups. Inverse-probability weighting was used to account for patient dropout.

**Results:**

Compared to non-users, prevalent users of metformin (beta 0.89, 95% *CI* 0.44; 1.33) and DPP-4i (0.72, 0.06; 1.37) experienced a slower cognitive decline with time. Secondly, compared to DPP-4i, the use of insulin (−1.00, −1.95; −0.04) and sulfonylureas (−1.19; −2.33; −0.04) was associated with larger point-wise decrements in MMSE with annual intervals.

**Conclusions:**

In this large cohort of patients with diabetes and dementia, the use of metformin and DPP-4i was associated with a slower decline in MMSE scores. Further examination of the cognitive effects of metformin and incretin-based medications is warranted.

**Supplementary Information:**

The online version contains supplementary material available at 10.1186/s13195-021-00934-0.

## Background

Diabetes is a complex risk factor for cognitive deterioration and a common comorbidity in dementia [1, 2]. In addition, multiple antidiabetic drugs have been evaluated in randomized and observational settings for putative cognitive properties. Specifically, metformin has been associated with protection against overall dementia risk and memory decline [3, 4]; however, some studies suggest an even opposite relationship [5], possibly due to vitamin B12 deficiency in long-term users [6] contributing to neurodegeneration.

Moreover, the role of cerebral insulin gained recognition in the last decade [7]; however, neither intranasal nor systemic insulin administration has significantly improved cognitive functioning [8, 9]. Similarly, the studies on sulfonylurea derivates have found either no association [10] or higher relative risk of dementia compared to metformin [11]. On the other hand, both sulfonylurea and systemic insulin treatment are associated with the risk of hypoglycemia [12], which may contribute to dementia incidence [13]. Moreover, manifest dementia is an independent predictor of severe hypoglycemia [14], as cognitive health is a prerequisite for proper diabetes self-management [15].

Thiazolidinediones (TZD) have not exhibited cognitive benefit in a randomized controlled setting [16]; moreover, faster memory decline was observed in TZD users among patients with Alzheimer’s disease (AD) [4].

Among the drugs affecting the incretin system, the glucagon-like peptide-1 analogues (GLP-1a) increased cerebral glucose metabolism in a small randomized controlled trial [17], while multiple neuroprotective mechanisms have been suggested in animal models of AD [18]. Second, the use of dipeptidyl-peptidase-4 inhibitors (DPP-4i) was cognitively protective in observational [4, 19] but not in interventional studies [20].

The potential recommendations for cognitive performance are further complicated by the applications of specific antidiabetic drugs in the course of diabetes (diabetes control, complications, application method, patient preference, etc.).

In summary, despite robust relationships between diabetes and dementia, it is unclear whether some antidiabetic medications provide cognitive benefit compared to others. Moreover, studies evaluating the rate of cognitive decline in patients with established dementia are scarce. Due to the absence of disease-modifying dementia treatment, good management of comorbidities can play a major role in preserving residual cognitive functioning in dementia patients.

The aim of this study was to compare the change in Mini-Mental State Examination (MMSE) scores among users of five antidiabetic drug groups in a large cohort of patients with diabetes and AD or mixed-pathology dementia (MixDem).

## Material and methods

This was a prospective open-cohort study with data originating from five Swedish registries/databases. Swedish personal identification number (personnummer) was used to merge information across data sources, with the National Board of Health and Welfare and Statistics Sweden performing the merge and data anonymization. Registers and data are described below.

### Swedish Dementia Registry (SveDem)

SveDem was established in 2007 with the aim to register all patients with dementia in Sweden at the time of diagnosis [21, 22]. SveDem comprises information on clinical determinants (e.g., MMSE), demography (e.g., age and living arrangements), community support (e.g., daycare), and common pharmacological treatment [21]. Dementia diagnoses are coded using ICD-10 and comprise AD, MixDem (both AD and vascular pathology present), vascular dementia, Lewy body dementias, frontotemporal dementia, unspecified, and other dementia types. Patients are followed up on an annual basis with clinical examination including cognitive assessment using MMSE.

#### Dementia

Out of the 80,004 patients registered in SveDem between May 1, 2007, and October 16, 2018, we included only patients with a diagnosis of AD or MixDem who were also diagnosed with diabetes (diabetes sections below) and had been followed up at least once. SveDem provided data on demography (age, sex), type of dementia, cohabitation, and baseline and follow-up dates with MMSE scores (the outcome). After applying sample restrictions, the final cohort consisted of 1873 patients with 4732 observations (baseline and follow-ups; supplementary figure 1). This population was then used to sample propensity-score exposure-matched pairs for the analyses.

### Swedish National Patient Register (Patient Register)

The Patient Register provided records on inpatient diagnoses since 1998 and specialized outpatient visits since 2001 [23] until December 31, 2017. Diagnoses were coded according to the 10th version of the International Classification of Diseases (ICD-10) [24].

#### Diabetes mellitus

Diagnosis of diabetes was determined when the ICD-10 codes E10–E14 occurred in the Patient Register or antidiabetic drug dispensation (Anatomical Therapeutic and Chemical [ATC] classification code A10) was observed in the Swedish Prescribed Drug Register (Drug Register) prior to and including the date of dementia diagnosis. Subsequently, diabetes was grouped into three types—type 1 diabetes, type 2 diabetes, and other/unspecified diabetes (for details on extraction and coding, see supplementary algorithm 1). Only patients with type 2 diabetes and other/unspecified types were included for the analyses.

Baseline diabetes duration was based on the difference between the date of dementia diagnosis and the date of the earliest record of diabetes—either in the Patient Register where the diagnosis of diabetes occurred or the earliest dispensation date of ATC code A10 from the Drug Register, whichever came first.

#### Comorbidities

To adjust for the effect of additional chronic diseases, we created a baseline comorbidity index as described by Charlson et al. [25], using the algorithm described by Quan et al. [26] as a weighted sum of diagnosed chronic disorders up to and including the date of dementia diagnosis. Renal disease was not included in the index and was extracted as a separate variable. Diabetes and dementia variables were omitted from the index.

### Longitudinal integrated database for health insurance and labour market studies (LISA)

LISA is an administrative database covering the adult Swedish population since 1990 and provides high-quality information on major socioeconomic characteristics (sick leave, disability, pensions, etc.) [27].

#### Disposable income

To take baseline socioeconomic position into account, disposable income at the time of dementia diagnosis inflated on the 2019 value of Consumer Price Index was extracted from LISA and categorized into low-, middle-, and high-income groups with 33rd and 66th percentiles used as cut-offs.

### Swedish Prescribed Drug Register

The Drug Register was established in 2005 and stores information on all dispensed drug prescriptions at Swedish pharmacies using ATC coding [28]. Medication dispensation data (=prescription fills) were extracted from the start of the register until December 31, 2018.

#### Diabetes mellitus

ATC codes A10 (drugs used in diabetes), A10A (insulins), and A10B (blood glucose-lowering drugs excluding insulin) before and after dementia diagnosis extracted from the Drug Register were used in combination with the Patient Register to identify overall diabetes prevalence and duration and to classify diabetes types (see supplementary algorithm 1).

#### Antidiabetic medications

Data on antidiabetic drug classes was extracted from the Drug Register according to the following ATC codes—insulin (A10A), metformin (A10BA02), sulfonylurea derivates (SU; A10BB), thiazolidinediones (A10BG), dipeptidyl-peptidase-4 inhibitors (A10BH), glucagon-like peptide-1 analogues (A10BJ), and sodium-glucose co-transporter-2 inhibitors (SGLT-2i; A10BK). Dispensations of the individual medication classes were extracted on a yearly basis prior to the date of dementia diagnosis date. GLP-1a and SGLT-2i could not be directly analyzed due to too few users; however, their use was matched on in the propensity-score matching. Two frameworks for exposure were created.

First, patients were grouped into baseline users and non-users of individual medications according to the incident and prevalent exposure design. A subject was an incident user if a dispensation of medication was observed in the 1-year period prior to and including the date of dementia diagnosis, without a record of medication dispensation prior to the 1-year period. Complementary incident non-user was a subject without a dispensation in neither the 1-year period nor at any time before the diagnosis of dementia. Prevalent use was based on the presence/absence of at least one medication dispensation prior to the diagnosis of dementia.

Second, we compared non-metformin antidiabetic medications directly. For example, to compare baseline insulin vs baseline sulfonylurea, patients exposed to insulin treatment at least once prior to diagnosis of dementia while never having sulfonylurea prior to diagnosis of dementia constituted one group, while subjects who had sulfonylurea and never had insulin constituted the comparison group. Due to the low number of possible pairs, we could only analyze prevalent users (see the section “Statistical analysis”). Metformin was not compared directly to other medications due to its specific position as first-line therapy and very frequent use in the cohort, thus lacking a sufficient number of patients in the never-metformin control group.

Subsequently, propensity-score matching on baseline exposure assignment was performed to produce comparable user/non-user and user/other-user pairs. Afterwards, the associations between antidiabetic drug usage and the change in post-dementia MMSE scores were assessed in intention-to-treat analyses.

#### Supplementary medication

Dispensations of antihypertensive (ATC codes C02, C07, C08, and C09), hypolipidemic (statins, C10AA), antithrombotic (B01), antipsychotic (N05A), antidepressant drugs (N06A), and cholinesterase inhibitors (N06DA) were extracted up to 3 years prior to and including the date of dementia diagnosis as recorded by the Drug Register.

### Swedish Cause of Death Register (Death Register)

The records in the Death Register begin from the year 1952 and are the basis for official statistics on causes of death in Sweden [29]. The register’s purpose is to describe the development of national all-cause and specific-cause mortality.

#### Mortality

We extracted the information from the Death Register since its initiation until October 16, 2018—the end of the study follow-up. Overall mortality was considered if a valid record (patient death dated after the date of dementia diagnosis) was present. The information on mortality was used to determine censoring and to account for patient dropout.

### Study sample selection

The original cohort consisted of 80,004 patients diagnosed with dementia and registered to SveDem until October 16, 2018. SveDem data were linked with other data sources and several selection criteria were applied, mainly excluding incorrect or missing baseline information. Afterwards, 12,396 patients who had diagnosis of diabetes and dementia at baseline were identified. After including only patients with at least one follow-up, exclusions of other dementia types than AD or MixDem, patients with diabetes type 1 and baseline severe dementia (MMSE < 10; to avoid floor effects), the final cohort consisted of 1873 patients and 4732 observations (supplementary figure 1).

### Statistical analysis

#### Propensity-score matching

From the whole cohort of 1873 subjects with diabetes and dementia, we sampled PS-matched comparable pairs of users/non-users and users/other-users of antidiabetic medications as per the framework described above. The 1:1 and 1:4 nearest-neighbor matching with 0.1 caliper of the logit of the propensity score was used. Baseline characteristics used to generate PS included age at dementia diagnosis; sex; cohabitation; dementia type; Charlson comorbidity score; renal disease; diabetes type and duration; income category; use of statins; antihypertensive, antithrombotic, antipsychotic, and antidepressant drugs; cholinesterase inhibitors; use of other glucose-lowering drugs apart from insulin  (as a summary measure user/non-user); and use of insulin (omitted in insulin analyses) prior to dementia diagnosis. Matching was less extensive in situations with few available exposed subjects (to uphold the 10 subjects per predictor rule), and matching priority was given to prognostic characteristics. Matching ratio 1:4 was used in the prevalent-user analyses of DPP-4i and TZD, all incident-user analyses, and comparisons of insulin vs DPP-4i, insulin vs TZD, sulfonylurea vs TZD, and sulfonylurea vs DPP-4i. Other cohorts were matched using a 1:1 ratio.

For the incident-user analyses, we identified the following user/non-user cohorts: 101 metformin users vs 277 non-users, 66 insulin users vs 263 non-users, and 37 sulfonylurea users vs 147 non-users. Prevalent-user analyses were based on 514 metformin user/non-user pairs, 543 insulin pairs, 640 sulfonylurea pairs, 67 TZD users vs 260 non-users, and 103 DPP-4i users vs 389 non-users (Table 1).

**Table 1:** Balance in baseline characteristics among prevalent users versus non-users of antidiabetic medications—propensity-score-matched cohorts

	Metformin Yes (*n*=514)	Metformin No (514)	*p*	*SMD*	DPP-4i Yes (103)	DPP-4i No (389)	*p*	*SMD*	Sulfonylurea Yes (640)	Sulfonylurea No (640)	*p*	*SMD*
Age	79.1 (6.3)	79.3 (6.5)	0.61	−0.03	77.8 (5.6)	77.6 (6.7)	0.80	0.05	79.1 (6.4)	79.1 (6.2)	0.90	0.01
Female	261 (50.8%)	253 (49.2%)	0.66	0.03	49 (47.6%)	54 (52.4%)	0.71		326 (50.9%)	324 (50.6%)	0.91	−0.01
Living alone	204 (39.7%)	204 (39.7%)	0.74	−0.01	34 (33.0%)	123 (31.6%)	0.96	−0.07	248 (38.8%)	244 (38.1%)	0.91	−0.02
Institutionalized	6 (1.2%)	9 (1.8%)			2 (1.9%)	8 (2.1%)			13 (2.0%)	15 (2.3%)		
Baseline MMSE	22 (5)	22 (6)	0.95		23 (5)	23 (5)	0.53		22 (6)	23 (5)	0.27	
AD	277 (53.9%)	298 (58.0%)	0.19	0.08	56 (54.4%)	222 (57.1%)	0.62		335 (52.3%)	348 (54.4%)	0.47	0.04
MixDem	237 (46.1%)	216 (42.0%)			47 (45.6%)	167 (42.9%)			305 (47.7%)	292 (45.6%)		
Diabetes duration	6.2 (6.3)	5.7 (6.1)	0.30	0.04	8.9 (4.8)	8.9 (4.7)	0.59	−0.01	7.9 (4.7)	8.1 (5.9)	0.88	−0.02
Charlson index	2 (2)	2 (2)	0.52	−0.03	2 (2)	1 (1)	0.37	0.07	2 (2)	2 (2)	0.52	0.01
Renal disease	22 (4.3%)	24 (4.7%)	0.76	−0.02	10 (9.7%)	23 (5.9%)	0.17	0.06	28 (4.4%)	30 (4.7%)	0.79	−0.01
Antihypertensives	398 (77.4%)	387 (75.3%)	0.42	0.06	87 (84.5%)	326 (83.8%)	0.87	−0.01	524 (81.9%)	518 (80.9%)	0.67	0.02
Statins	351 (68.3%)	329 (64.0%)	0.15	0.10	91 (88.3%)	273 (70.2%)	<0.001		468 (73.1%)	461 (72.0%)	0.66	0.02
Antithrombotics	366 (71.2%)	350 (68.1%)	0.28	0.07	79 (76.7%)	273 (70.2%)	0.19		466 (72.8%)	464 (72.5%)	0.90	0.01
Antipsychotics	21 (4.1%)	14 (2.7%)	0.23	0.08	4 (3.9%)	11 (2.8%)	0.53	0.06	12 (1.9%)	12 (1.9%)	1.00	0.00
Antidepressants	158 (30.7%)	162 (31.5%)	0.79	−0.02	26 (25.2%)	98 (25.2%)	0.99	0.01	190 (29.7%)	188 (29.4%)	0.90	0.01
ChEI	127 (24.7%)	128 (24.9%)	0.94	−0.01	20 (19.4%)	85 (21.9%)	0.59	−0.04	144 (22.5%)	150 (23.4%)	0.69	−0.02
Other GLDs	162 (31.5%)	171 (33.3%)	0.55	0.04	98 (95.1%)	324 (83.3%)	0.002		492 (76.9%)	479 (74.8%)	0.40	0.05
Insulin	161 (31.3%)	150 (29.2%)	0.46	0.04	52 (50.5%)	149 (38.3%)	0.03		268 (41.9%)	256 (40.0%)	0.50	0.04
Income												
Low	171 (33.3%)	179 (34.8%)	0.49	−0.01	28 (27.2%)	112 (28.8%)	0.87	−0.01	226 (35.3%)	222 (34.7%)	0.95	−0.01
Middle	175 (34.0%)	157 (30.5%)			35 (34.0%)	122 (31.4%)			196 (30.6%)	201 (31.4%)		
High	168 (32.7%)	178 (34.6%)			40 (38.8%)	155 (39.8%)			218 (34.1%)	217 (33.9%)		
Total eligible	1341 (38.3%)	532 (96.6%)			103 (100%)	1770 (22.0%)			664 (96.3%)	1209 (52.9%)		
	Insulin Yes (543)	Insulin No (543)	*p*	*SMD*	TZD Yes (67)	TZD No (260)	*p*	*SMD*				
Age	77.9 (6.9)	78.2 (6.4)	0.51	−0.04	76.3 (5.9)	76.4 (6.4)	0.91	0.01				
Female	271 (49.9%)	272 (50.1%)	0.76	−0.02	40 (59.7%)	142 (54.6%)	0.46					
Living alone	196 (36.1%)	193 (35.5%)	0.29	0.01	13 (19.4%)	53 (20.4%)	0.68	−0.04				
Institutionalized	14 (2.6%)	7 (1.3%)			1 (1.5%)	9 (3.5%)						
Baseline MMSE	23 (6)	23 (5)	0.97		22 (5)	23 (6)	0.84					
AD	295 (54.3%)	303 (55.8%)	0.63	0.03	44 (65.7%)	154 (59.2%)	0.34					
MixDem	248 (45.7%)	240 (44.2%)			23 (34.3%)	106 (40.8%)						
Diabetes duration	8.3 (4.7)	8.4 (5.0)	0.85	0.01	8.3 (4.9)	8.7 (5.1)	0.99	−0.01				
Charlson index	2 (2)	2 (2)	0.55	−0.02	1 (1)	1.5 (1)	0.74	−0.02				
Renal disease	24 (4.4%)	23 (4.2%)	0.88	0.01	2 (3.0%)	11 (4.2%)	0.64					
Antihypertensives	438 (80.7%)	433 (79.7%)	0.70	0.02	56 (83.6%)	216 (83.1%)	0.92					
Statins	404 (74.4%)	394 (72.6%)	0.49	0.04	50 (74.6%)	196 (75.4%)	0.90					
Antithrombotics	399 (73.5%)	395 (72.7%)	0.78	0.02	44 (65.7%)	183 (70.4%)	0.46					
Antipsychotics	18 (3.3%)	19 (3.5%)	0.87	−0.01	2 (3.0%)	7 (2.7%)	1.00					
Antidepressants	182 (33.5%)	187 (34.4%)	0.75	−0.02	21 (31.3%)	82 (31.5%)	0.98					
ChEI	132 (24.3%)	141 (26.0%)	0.53	−0.04	16 (23.9%)	59 (22.7%)	0.84	0.01				
Other GLDs	459 (84.5%)	455 (83.8%)	0.74	0.02	65 (97.0%)	249 (95.8%)	0.64	0.07				
Insulin					32 (47.8%)	110 (42.3%)	0.42					
Income												
Low	190 (35.0%)	195 (35.9%)	0.73	−0.01	22 (32.8%)	83 (31.9%)	0.98					
Middle	167 (30.8%)	155 (28.5%)			21 (31.3%)	81 (31.2%)						
High	186 (34.3%)	193 (35.5%)			24 (35.8%)	96 (36.9%)						
Total eligible	645 (84.2%)	1228 (44.2%)			68 (99.0%)	1805 (14.4%)						

Baseline characteristics were compared per baseline prevalent exposure assignment—users at any time prior to dementia diagnosis; *p*-values refer to the exposure “Yes” vs exposure “No” comparisons. Age is described as mean (*SD*); Charlson comorbidity index, diabetes duration, and MMSE are described as median (*IQR*); all other variables are described as *n* (%); *SMD*s were calculated for the matching variables; DPP-4i and TZD exposure groups were matched using 1:4 ratio; “Total eligible” expresses the number of eligible subjects for propensity-score matching from the original cohort, with % retained after PS matching

*AD* Alzheimer’s disease, *ChEI* cholinesterase inhibitors, *DPP-4i* dipeptidyl-peptidase-4 inhibitors, *MixDem* mixed-pathology dementia, *MMSE* Mini-Mental State Examination, *GLDs *glucose-lowering drugs apart from insulin, *SMD* standardized mean differences, *TZD* thiazolidinediones

In the non-metformin antidiabetic drug comparisons, 259 pairs of insulin vs sulfonylurea users were generated, 138 insulin vs 35 TZD users, 199 insulin vs 51 DPP-4i users, 111 sulfonylurea vs 31 TZD users, 141 sulfonylurea vs 38 DPP-4i users, and 45 DPP-4i vs TZD pairs. The baseline differences between matched cohorts as well as the number eligible for individual matchings from the original cohort are summarized in Table 1 and supplementary tables 1 and 3.

#### Patient dropout—inverse-probability weighting

Patient dropout was determined, if the patient had neither died nor did the study end occur in the year following the last observed MMSE. The last observed MMSE was defined as the last observed MMSE measurement, provided the time difference between the last observed MMSE examination and previous MMSE examination was more than 9 months to reflect the yearly schedule of follow-ups. If the difference was less than 9 months, the 1-year period for dropout was assessed from the previous date of MMSE measurement. Using this definition, the overall dropout rate was high (1382 patients [73.8%]; supplementary table 2); thus, we weighted the analyses using inverse probability of remaining in the study based on the MMSE scores in the previous observation and time since baseline. We used the same process as described by Handels and colleagues [30]: first computing the probability of dropout in the next observation, then defining cumulative probabilities of remaining in the study, and finally producing an inverse of the cumulative probabilities—inverse-probability weights of remaining in the study. The weights were unstabilized and truncated at 100 if the weight was above the 99th percentile of the weight distribution to avoid the models being dominated by few large weights.

#### Descriptive statistics

Differences in baseline characteristics between the matched pairs of users and non-users of antidiabetic medications were assessed using chi-square, independent samples *t*-test, and their non-parametric equivalents. Standardized mean differences (*SMD*s) were used to assess balance in the propensity-score-matched cohorts.

#### MMSE change—mixed-effects models

In the PS-matched cohorts, random-slope linear mixed-effects models were used to determine the beta coefficients with 95% confidence intervals of the associations between the use of antidiabetic medications and change in MMSE scores with time in years. Fixed effects in the model included only the interaction between the users/non-users or users/other users of antidiabetic medication with time. Random effects were included for subjects and time (intercept and slope). Three mixed-effects models were designed in the propensity-score-matched cohorts—unweighted analysis, inverse-probability-weighted analysis, and weighted analysis in the imputed dataset. Residual baseline differences between the matched cohorts were adjusted for and kept in the models if both the association with MMSE and 10% change in the main exposure were significant. Subjects with missing information in matching or weighting variables were excluded prior to analysis (see supplementary figure 1).

Data were analyzed using Stata v16 (Stata Statistical Software: Release 16. StataCorp LLC, College Station, TX) and R version 4.0.0 [31] with package “MatchIt.”

#### Sensitivity analysis—imputed dataset

To model the ideal scenario with yearly follow-ups, we have imputed MMSE scores based on the slope between the observed MMSE measurements and time from baseline. Missing scores were imputed using multivariate multiple imputation with chained equations. Imputations were done only in observations with missing yearly follow-ups between observed MMSE (e.g., observed baseline and year 3) and not performed beyond observed MMSE. Afterwards, the inverse-probability-weighted mixed-effects models were performed in the imputed dataset.

## Results

### Baseline differences

The differences between the matched cohorts are summarized in Table 1 (prevalent use) and supplementary table 1 (incident use) and 3 (drug vs drug comparisons).

The PS matching provided balanced exposure groups, except for few differences. DPP-4i users were more frequently exposed to statins in all cohorts (88.3% vs 70.2% in prevalent users; 90.2% vs 75.9% in DPP-4i vs insulin; 97.4% vs 75.2% DPP-4i vs sulfonylurea). Incident insulin users were less commonly treated with antidepressants (15.2% vs 34.6%). Finally, incident metformin users had significantly shorter diabetes duration (0.8 vs 3.5 years). All residual differences were adjusted for in the mixed models.

### MMSE and antidiabetic medications

The results from the random-slope linear mixed-effects models are summarized in Tables 2 and 3. In the weighted user vs non-user analyses, the use of metformin was associated with a slower annual decline in MMSE compared to non-users, specifically 0.89 points slower in prevalent users vs non-users (*β* 0.89, *95% CI* 0.44; 1.33), however not in incident-user weighted analysis (0.70, −0.16; 1.56). Similar association was observed in the unweighted (0.67, 0.35; 0.99) and imputed analyses (0.88, 0.44; 1.32). The prevalent use of DPP-4i was associated with a slower annual MMSE decline compared to non-users in the weighted (0.72, 0.06; 1.37) and imputed (0.70, 0.03; 1.37) analyses, but not statistically significantly in the unweighted analysis (0.53, −0.02; 1.09; *p* = 0.06).

**Table 2:** Annual point change in post-dementia Mini-Mental State Examination scores associated with the use of antidiabetic medications

	Unweighted analysis User vs non-user			Weighted analysis User vs non-user			Imputed weighted analysis User vs non-user		
	*β* coefficients (*95% CI*)	*Z*	*p*	*β* coefficients (*95% CI*)	*Z*	*p*	*β* coefficients (*95% CI*)	*Z*	*p*
Propensity-score-matched cohorts									
Metformin x time (incident users)	0.38 (−0.20; 0.95)	1.28	0.20	0.70 (−0.16; 1.56)	1.59	0.11	0.53 (−0.31; 1.37)	1.23	0.22
Metformin x time (prevalent users)	0.67 (0.35; 0.99)	4.15	<0.001	0.89 (0.44; 1.33)	3.93	<0.001	0.88 (0.44; 1.32)	3.91	<0.001
Insulin x time (incident users)	−0.25 (−0.83; 0.33)	−0.84	0.40	−0.28 (−1.22; 0.67)	−0.57	0.57	−0.37 (−1.34; 0.60)	−0.74	0.46
Insulin x time (prevalent users)	−0.06 (−0.37; 0.25)	−0.41	0.68	−0.03 (−0.48; 0.42)	−0.13	0.90	−0.12 (−0.56; 0.32)	−0.54	0.59
Sulfonylurea x time (incident users)	0.05 (−0.84; 0.93)	0.10	0.92	−0.10 (−1.38; 1.17)	−0.16	0.87	−0.19 (−1.48; 1.11)	−0.28	0.78
Sulfonylurea x time (prevalent users)	−0.08 (−0.36; 0.20)	−0.55	0.59	−0.11 (−0.53; 0.31)	−0.52	0.60	−0.13 (−0.56; 0.29)	−0.62	0.53
TZD x time (prevalent users)	0.12 (−0.49; 0.73)	0.38	0.71	0.14 (−0.76; 1.04)	0.30	0.76	0.04 (−0.91; 0.98)	0.07	0.94
DPP-4i x time (prevalent users)	0.53 (−0.02; 1.09)	1.64	0.06	0.72 (0.06; 1.37)	2.14	0.032	0.70 (0.03; 1.37)	2.04	0.041

Beta coefficients were acquired from random-slopes linear mixed-effects models. Weighting was performed for the inverse probability of remaining in the study. Sulfonylurea and DPP-4i incident-user analyses and GLP-1a and SGLT-2i prevalent-user analyses were performed in 1:4 PS-matched cohorts. Time scale is expressed in years

*DPP-4i* dipeptidyl-peptidase-4 inhibitors, *GLP-1a* glucagon-like peptide-1 analogues, *SGLT-2i* sodium-glucose co-transporter-2 inhibitors

**Table 3:** Comparisons between non-metformin antidiabetic drugs and annual point change in Mini-Mental State Examination scores

	Unweighted analysis Drug vs drug			Weighted analysis Drug vs drug			Imputed weighted analysis Drug vs drug		
	*β* coefficients (*95% CI*)	*Z*	*p*	*β* coefficients (*95% CI*)	*Z*	*p*	*β* coefficients (*95% CI*)	*Z*	*p*
Propensity-score-matched cohorts									
Insulin vs sulfonylurea x time (259 user pairs)	0.14 (−0.28; 0.56)	0.65	0.52	0.29 (−0.35; 0.92)	0.89	0.37	0.29 (−0.32; 0.90)	0.93	0.35
Insulin vs TZD x time (138 users vs 35 users)	−0.23 (−1.04; 0.58)	−0.56	0.58	−0.86 (−1.95; 0.24)	−1.53	0.13	−0.90 (−2.03; 0.23)	−1.56	0.12
Insulin vs DPP-4i x time (199 users vs 51 users)	−0.82 (−1.72; 0.07)	−1.80	0.07	−1.00 (−1.95; −0.04)	−2.04	0.042	−0.93 (−1.87; 0.01)	−1.94	0.053
Sulfonylurea vs DPP-4i x time (141 users vs 38 users)	−1.07 (−2.10; −0.05)	−2.05	0.040	−1.19 (−2.33; −0.04)	−2.05	0.041	−1.15 (−2.33; 0.02)	−1.93	0.054
Sulfonylurea vs TZD x time (111 users vs 31 users)	−0.62 (−1.58; 0.34)	−1.26	0.21	−1.00 (−2.34; 0.35)	−1.46	0.15	−0.98 (−2.34; 0.38)	−1.41	0.16
DPP-4i vs TZD x time (45 user pairs)	0.15 (−0.78; 1.08)	0.31	0.76	−0.19 (−1.48; 1.10)	−0.29	0.77	−0.16 (−1.48; 1.15)	−0.25	0.81

Based on random-slopes linear mixed-effects models. Medication use was assigned, if a patient had a dispensation of the primary medication prior to diagnosis of dementia, while never having a dispensation of the compared medication prior to diagnosis of dementia and vice versa. Weighting was performed for the inverse probability of remaining in the study. Time was expressed in years

**Fig. 1:**
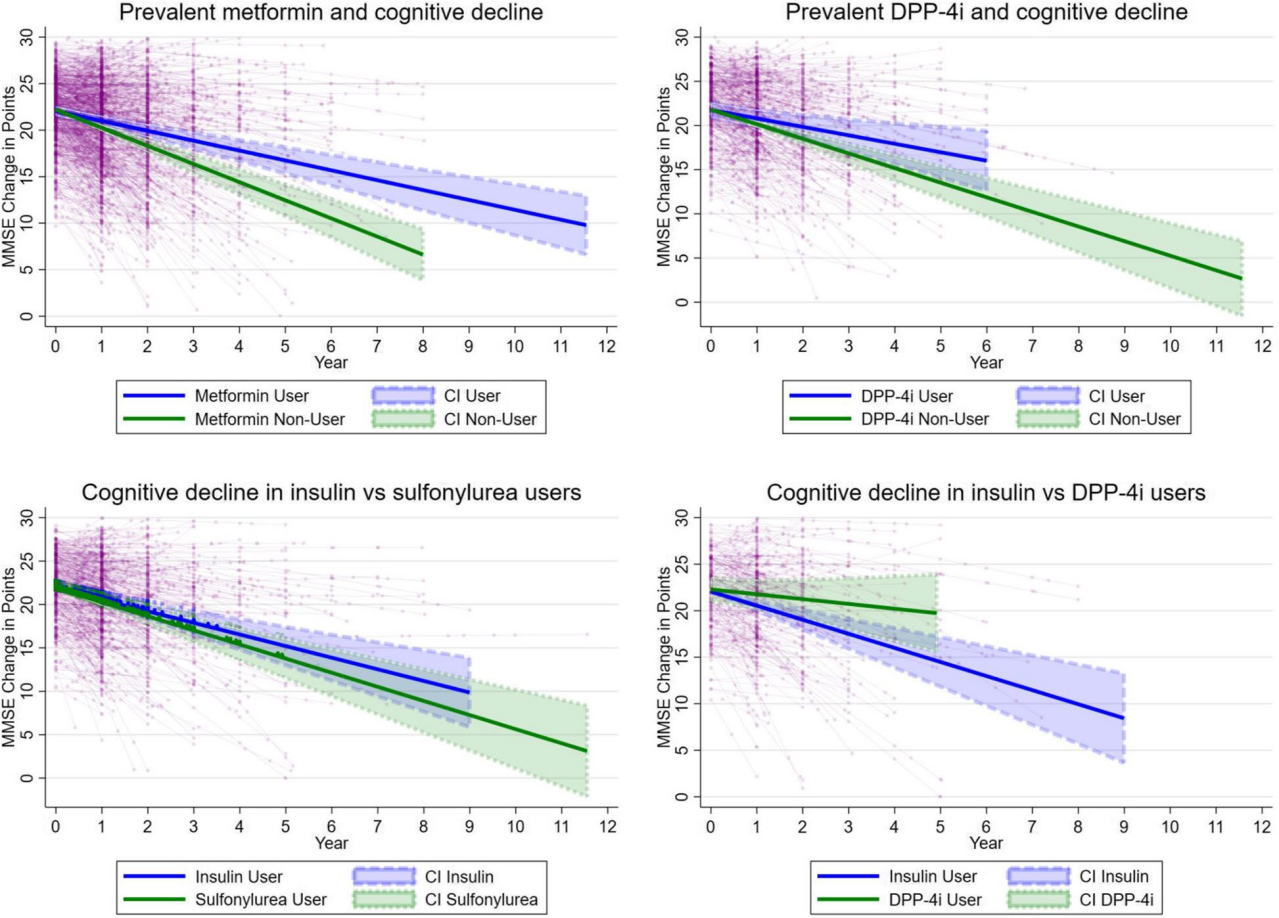
Linear changes in MMSE scores associated with the use of metformin, DPP-4i, insulin, and sulfonylurea exposure among patients with dementia and diabetes. Figures are based on weighted prevalent-user analyses; DPP-4i, dipeptidyl-peptidase-4 inhibitors; CI, confidence interval; MMSE, Mini-Mental State Examination; year represents time after baseline; thin lines represent MMSE change in individual subjects (circles) in time, and thick lines represent estimated MMSE change derived from the mixed models

Figure 1 visualizes the main observed trends over time in the annual MMSE decline in users of metformin, DPP-4i, and comparisons between insulin and sulfonylurea and insulin and DPP-4i based on the weighted analyses.

In the head-to-head comparisons of the non-metformin antidiabetic medications, the use of sulfonylureas was associated with an accelerated MMSE decline compared to DPP-4i users (−1.10, −2.12; −0.08; weighted analyses), with similar findings in the unweighted (−1.07; −2.10; −0.05) and imputed models (−1.07, −2.12; −0.02). Similarly, insulin users experienced an accelerated cognitive decline compared to DPP-4i users (−1.00, −1.95; −0.04) when weighted for dropout.

## Discussion

In this large cohort of patients with diabetes and dementia, the use of metformin and DPP-4i was associated with a slower decline in MMSE scores in time compared to non-users. Moreover, patients with dementia using DPP-4i experienced a slower decline in MMSE scores compared to sulfonylurea and insulin users.

The overall (prevalent) use of metformin was associated with a slower decline in MMSE compared to non-users, suggesting either a well-managed diabetes in metformin users or a direct neuroprotective effect of metformin. While lower dementia risk was observed in metformin users in multiple studies [11, 32, 33], MMSE was unaffected in a 2018 meta-analysis [3]. On the other hand, metformin was recently associated with protection against memory decline, however only in patients with normal cognition and without ApoE-ε4 burden [4]. While we could not stratify on ApoE genotype, we analyzed metformin initiation, which failed to provide significant benefit to users, despite the shorter diabetes duration. Metformin’s role in the AD pathological process is uncertain, with both neuroprotective [34–36] and pathology-accelerating properties [37, 38] reported in non-human studies. Recently, Wu and colleagues suggested that metformin’s putative neuroprotection may be limited to preclinical AD stages, whereas its later use may contribute to AD pathology [4]. In our cohort, we conclude metformin’s cognitive effect as neutral to positive; however, we lacked a comparable number of never-exposed to metformin for prevalent users, and this analysis drove the protective association. Moreover, the comparison group of metformin non-users comprised a non-negligible number of subjects without any medication for diabetes, which likely affected the association even after thorough matching; however, it is not clear in which direction. Possibly, metformin’s molecular changes may not be directly reflected on a robust point-based MMSE score; however, if metformin would exhibit serious cognitive harm, this would likely be noticed between the follow-ups. Overall, a life-course approach combined with the evaluation of individual cognitive domains would be valuable to deconstruct metformin’s neuronal properties.

Importantly, the prevalent users of DPP-4i experienced a significantly slower decline in MMSE scores compared to non-users. Among incretin-based therapies, DPP-4i are frequently used in Swedish patients with dementia [39], possibly due to the oral application and neutral weight effects. In observational studies, DPP-4i were associated with a lower risk of dementia [10, 40] and improvements in immediate and delayed memory in patients with established AD [4]. On the other hand, a recent randomized trial of linagliptin failed to show cognitive benefit in high-risk albeit dementia-free type 2 diabetes patients [20].

In our cohort, DPP-4i exhibited a 0.7-point slower MMSE decline in users vs non-users, corroborating the findings by Isik and colleagues [19]. Similarly, Rizzo and colleagues concluded higher MMSE among DPP-4i-metformin therapy compared to sulfonylurea-metformin after 2 years [41]; however, patients with dementia were excluded making the comparison difficult. Moreover, the addition of DPP-4i vildagliptin to metformin was associated with higher MMSE at 6 months compared to metformin alone, but the study was underpowered for multivariate analyses [42].

DPP-4i users specifically experienced a slower MMSE decline compared to sulfonylurea and insulin users. Conceptually, patients using sulfonylureas and insulin may have been at greater risk of hypoglycemia (DPP-4i have negligible hypoglycemia risk) [12]; unfortunately, we could not test such hypothesis. On the other hand, Cukierman-Yaffe and colleagues have recently contested the connection between severe hypoglycemia and cognitive decline; however, it is unclear whether this extends to dementia patients [43].

DPP-4i exhibit their antidiabetic effect in a glucose-dependent manner by increasing the endogenous GLP-1 through inhibition of its breakdown [12]; however, their potency is lower compared to GLP-1a [44]. Interestingly, the preclinical studies on GLP-1’s role in cerebral metabolism concluded amelioration of oxidative stress, growth factor, and insulin signaling in AD pathology [18]. DPP-4i could possibly provide direct neuronal benefit; however, enhancing cerebral signaling through increasing the pool of GLP-1 is more likely [45] as the DPP-4i transfer through the blood-brain barrier is limited in comparison to GLP-1a [46].

Unfortunately, our study lacked the power to analyze GLP-1a users, which would strengthen the argument for the incretin-based therapies if a similar association was observed.

In general, the available evidence suggests that the protection associated with incretin therapies may be limited to later phases of neurodegeneration.

Despite the encouraging results for metformin and DPP-4i, the inter-follow-up MMSE change was rather modest (approximately 1 point) and the minimal clinically important decline in MMSE was recently estimated at around 2–3 points [47]. If accurate, the difference in DPP-4i and metformin users vs non-users should be observable approximately at the third-year follow-up. On the other hand, any preservation of cognitive functioning associated with the use of established antidiabetic drugs should be considered valuable, especially in the absence of disease-modifying therapy.

Neither sulfonylurea nor insulin was associated with a significant change in MMSE scores compared to non-users, while a similar decline was observed when directly comparing the two drugs or compared to TZD.

Sulfonylurea’s neurophysiological properties are not entirely clear, as some evidence suggests a higher risk of dementia in comparison to metformin [11, 32], or no association [10, 48]. In cognitive functioning, sulfonylureas have not modified either delayed or immediate memory [4], while a larger, albeit inconsistent global cognitive change was observed in sulfonylurea users in a pooled meta-analysis [48]. We provide robust evidence that sulfonylurea use does not affect global cognition in patients with AD or MixDem.

On the other hand, insulin treatment has a specific position in the treatment of type 2 diabetes in Sweden, where its use is prioritized in the guidelines [49] and common even in patients with dementia [39]. While insulin use was associated with a higher risk of all-cause dementia and a modestly larger decline in global cognition [48], the association was not found in other studies [9, 10]. In line with our findings, Cukierman-Yaffe and colleagues reported no effect of basal insulin on MMSE scores; however, the population was younger and cognitively unimpaired at baseline [9]. Thus, insulin indeed may be cognitively neutral; however, further research is needed to clarify if hypoglycemia may balance out some cognitive protection, especially in a region with frequent insulin usage.

We have not observed any substantial effect on global cognition in patients using TZD, corroborating the randomized controlled trials on rosiglitazone [16] and pioglitazone [50]. While TZD may accelerate memory decline in ApoE-ε4 non-carriers with AD and diabetes [4], we did not have access to such data. Currently, the only approved TZD in Europe is pioglitazone; however, a careful patient selection is necessary prior to drug initiation due to a higher risk of edema, heart failure, and fractures [12]. Overall, the use of TZD in elderly patients with dementia exhibited neither cognitive protection nor harm in our cohort.

In conclusion, metformin and DPP-4i had a positive effect on global cognition measured by MMSE in patients with diabetes and dementia. Further research should focus on the putative neurocognitive properties of incretin and biguanide therapy.

### Limitations

One of the main limitations is the observational nature of the study, precluding us from concluding causal relationships. Second, SveDem is a real-world database and thus suffers from significant patient dropout, which cannot be controlled for in the data collection phase. Conversely, we have addressed this issue of selective dropouts by weighting on the inverse probability of remaining in the study, which produced reasonable estimates and was previously used in the SveDem setting [30]. MMSE has known ceiling and floor effects; however, the median baseline MMSE score was 21 points (18 and 24 being the 25th and 75th percentile) and patients with baseline MMSE below 10 points were excluded. Most patients had been living with diabetes for several years; thus, there were insufficient incident-users for more recent medications and for head-to-head drug comparisons. While we had a plethora of data on confounding, information on two principal confounders was lacking—glycemic markers (such as HbA1c) and the ApoE genotype. The absence of HbA1c likely skewed some of the results in favor of metformin—being the first-line therapy and representing a group of patients with an overall shorter course of diabetes and fewer complications. On the other hand, the insufficient data on hypoglycemic episodes may have confounded the results of insulin and sulfonylureas. Secondly, the ApoE genotype information was lacking; thus, associations pertinent only to patients carrying ε4 genotype could have been diluted. Lastly, we had no information on the reason for medication dispensation and no access to further blood or CSF biochemistry that would supplement the matching strategy; thus, residual confounding or confounding by indication cannot be ruled out.

The study’s main strength comprises the large cohort of patients with diabetes and established dementia, a typically understudied multimorbid population. Moreover, high-quality data were available on medication dispensations allowing multiple exposure models and overall good coverage in the data sources [21, 23, 27–29]. In addition, the breadth of variables allowed extensive PS matching possibilities, decreasing the variability between matched subjects. This was observed even when matching was restricted and the differences in most unmatched variables were balanced as well. Importantly, the inclusion of incident-user and prevalent-user analyses, drug vs drug comparisons, and inverse-probability weighting provided comprehensive information about the use of antidiabetics and their association to a well-established global cognitive screening test—MMSE.

## Conclusions

In conclusion, there are significant differences between the common antidiabetic medications and their association with changes in MMSE scores. Specifically, metformin and the incretin-based medications should be further evaluated for possible cognitive properties.

## Supplementary Information

**Additional file 1: Supplementary Algorithm 1.** Diabetes types assessed from the Patient and Drug Register data.

**Additional file 2: Supplementary Figure 1.** Study sample selection. DPP-4i, dipeptidyl-peptidase-4 inhibitors; IPW, inverse-probability weighting; MMSE, Mini-Mental State Examination; SveDem, Swedish Dementia Registry; TZD, thiazolidinediones.

**Additional file 3: Supplementary Table 1.** Balance in baseline characteristics among incident users versus non-users of antidiabetic medications – propensity-score matched cohorts. ChEI, cholinesterase inhibitors; DPP-4i, dipeptidyl-peptidase-4 inhibitors; MMSE, Mini-Mental State Examination; GLDs, glucose-lowering drugs apart from insulin; SMD, standardized mean differences; Comparisons per baseline incident exposure assignment – exposure assessed at one-year period prior to dementia diagnosis in subjects without history of exposure before the one-year period; p-values refer to the exposure “Yes” vs exposure “No” comparisons; Age is described as mean (SD); Charlson comorbidity index, Diabetes duration and MMSE are described as median (IQR); all other variables are described as n (%); SMDs were calculated for the matching variables; All cohorts matched using 1:4 ratio; “Total eligible” expresses the number of eligible subjects for propensity-score matching from the original cohort, with % retained after PS matching.

**Additional file 4: Supplementary Table 2.** Dropout rate and number of follow-ups in observed and imputed data by users of antidiabetic medications. DPP-4i, dipeptidyl-peptidase-4 inhibitors; TZD, thiazolidinediones; Dropout was counted when the patient had neither died nor did the study end occur in the year following the last observed MMSE; Number of follow-ups are expressed in number of follow-ups in total per drug user, and percentage from baseline number of users; Imputed follow-ups were based on the slope of change between observed MMSE measurements and time from baseline.

**Additional file 5: Supplementary Table 3.** Baseline differences in the propensity-score matched comparison pairs of the non-metformin antidiabetic drug users. AD, Alzheimer’s disease; ChEI, cholinesterase inhibitors; DPP-4i, dipeptidyl-peptidase-4 inhibitors; MixDem, mixed-pathology dementia; MMSE, Mini-Mental State Examination; GLDs, glucose-lowering drugs apart from insulin; SMD, standardized mean differences; SMDs were calculated for the matching variables; Matching was restricted by number of available users; Insulin vs sulfonylurea analysis was matched using 1:1 ratio, otherwise 1:4 ratio was used; “Total eligible” expresses the number of eligible subjects for propensity-score matching from the original cohort, with % retained after PS matching.

## Data Availability

No data are available. The entities responsible for the original data and the Swedish law do not allow for sharing of the data from the Swedish national or quality-of-care registers.

## Abbreviations

AD: Alzheimer’s disease
ATC: Anatomical Therapeutic and Chemical
DPP-4i: Dipeptidyl-peptidase-4 inhibitors
GLP-1a: Glucagon-like peptide-1 analogues
LISA: Longitudinal integrated database for health insurance and labour market studies
MMSE: Mini-Mental State Examination
MixDem: Mixed-pathology dementia
GLD: Glucose-lowering drugs
SGLT-2i: Sodium-glucose co-transporter-2 inhibitors
*SMD*s: Standardized mean differences
SveDem: Swedish Dementia Registry
Patient Register: Swedish National Patient Register
Death Register: Swedish Cause of Death Register
TZD: Thiazolidinediones
