# Supplementary material for: The association of antidiabetic medications and Mini-Mental State Examination scores in patients with diabetes and dementia
Source: Alzheimers Res Ther. 2021 Dec 2;13:197. doi: 10.1186/s13195-021-00934-0 (PMC8641148; doi:10.1186/s13195-021-00934-0)
Supplement: Supplementary file 1 — Additional file 1: Supplementary Algorithm 1. Diabetes types assessed from the Patient and Drug Register data. [file 13195_2021_934_MOESM1_ESM.docx]

Supplementary algorithm 1. Diabetes types assessed from the Patient and Drug Register data

Type 1 diabetes was assigned if:

1. Patient had ICD-10 code E10 in the Patient Register, no other ICD-10 code relevant to diabetes (E11-E14) and had been on insulin-only antidiabetic treatment as recorded in the Drug Register. (“Definite type 1”)
2. Patient had ICD-10 code E10 in the Patient Register, as well as other relevant ICD-10 diabetes code (E11-E14), the ICD-10 code E10 was recorded prior to the other diabetes codes, and patient had been on insulin-only antidiabetic treatment as recorded in the Drug Register. (“Probable type 1”)

Type 2 diabetes was assigned if:

1. Patient had ICD-10 code E11 in the Patient Register and no other relevant ICD-10 diabetes code was recorded. (“Definite type 2”)
2. Patient had ICD-10 code E11 in the Patient Register as well as relevant ICD-10 diabetes codes for type 1 and other/unspecified diabetes, and ICD-10 code E11 was recorded prior to the other diabetes codes. (“Probable type 2”)
3. Patient had ICD-10 code E11 in the Patient Register as well as relevant ICD-10 diabetes codes for type 1 diabetes (E10), and ICD-10 code E11 was recorded prior to the ICD-10 code E10, and patient was not on insulin treatment as recorded in the Drug Register. (“Probable type 2”)
4. Patient had ICD-10 code E11 in the Patient Register as well as relevant ICD-10 diabetes codes for other/unspecified diabetes (E12-E14), and ICD-10 code E11 was recorded prior to the other diabetes codes. (“Probable type 2”)

Other/unspecified diabetes was assigned if:

1. Patient had not fulfilled criteria for type 1 or type 2 diabetes.

All patients with type 1 diabetes were excluded (both “definite” and “probable” types). Then, 3,077 (61.8%) patients were assigned with type 2 diabetes diagnosis (52.6% “Definite type 2”; 9.2% “Probable type 2”) and 1,903 (38.2%) were diagnosed with other/unspecified diabetes.
