# Supplementary material for: The association of antidiabetic medications and Mini-Mental State Examination scores in patients with diabetes and dementia
Source: Alzheimers Res Ther. 2021 Dec 2;13:197. doi: 10.1186/s13195-021-00934-0 (PMC8641148; doi:10.1186/s13195-021-00934-0)
Supplement: Supplementary file 2 — Additional file 2: Supplementary Figure 1. Study sample selection. DPP-4i, dipeptidyl-peptidase-4 inhibitors; IPW, inverse-probability weighting; MMSE, Mini-Mental State Examination; SveDem, Swedish Dementia Registry; TZD, thiazolidinediones. [file 13195_2021_934_MOESM2_ESM.docx]

Supplementary figure 1. Study sample selection
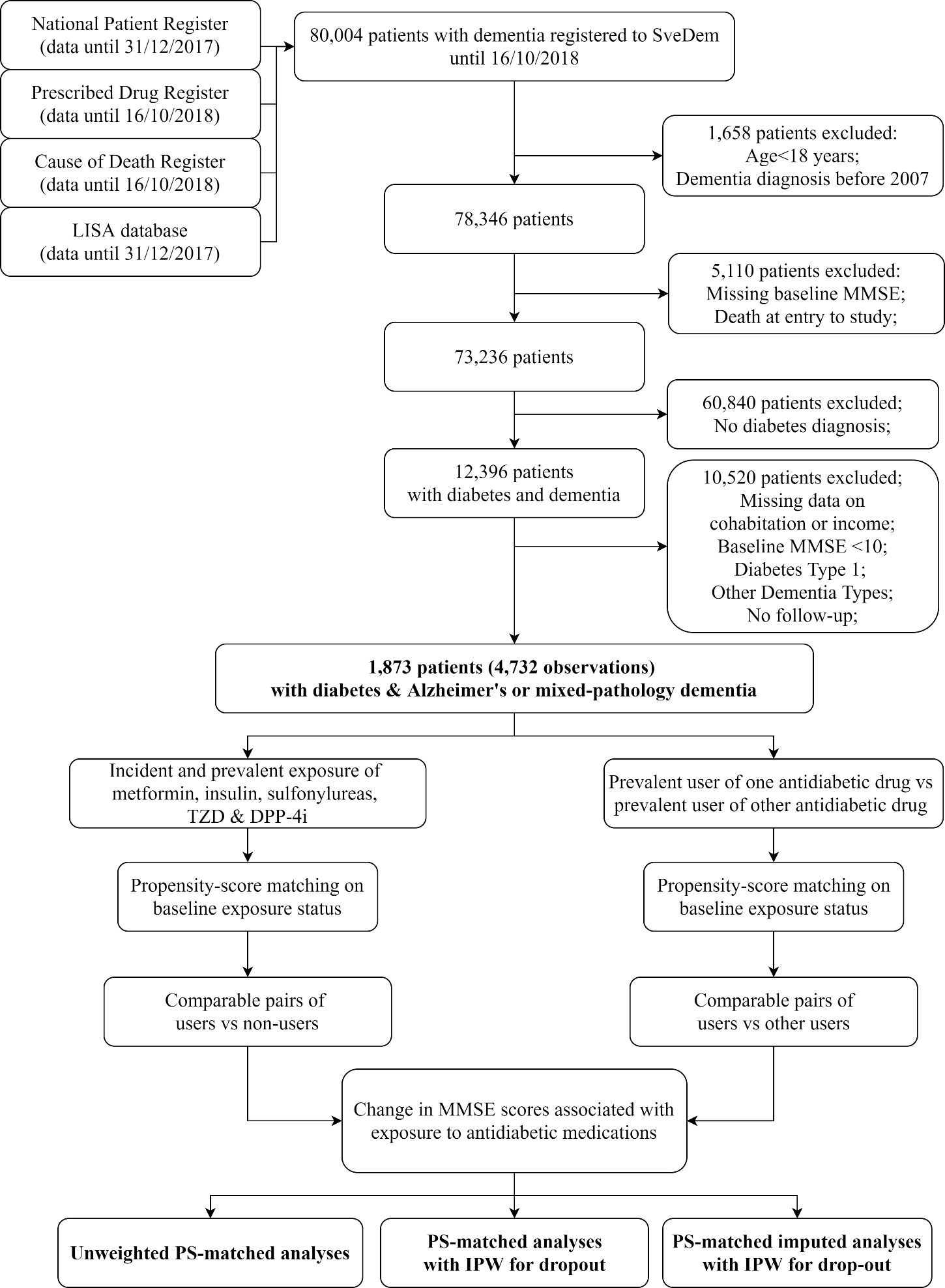
DPP-4i, dipeptidyl-peptidase-4 inhibitors; IPW, inverse-probability weighting; MMSE, Mini-Mental State Examination; SveDem, Swedish Dementia Registry; TZD, thiazolidinediones
