# Supplementary material for: The association of antidiabetic medications and Mini-Mental State Examination scores in patients with diabetes and dementia
Source: Alzheimers Res Ther. 2021 Dec 2;13:197. doi: 10.1186/s13195-021-00934-0 (PMC8641148; doi:10.1186/s13195-021-00934-0)
Supplement: Supplementary file 3 — Additional file 3: Supplementary Table 1. Balance in baseline characteristics among incident users versus non-users of antidiabetic medications – propensity-score matched cohorts. ChEI, cholinesterase inhibitors; DPP-4i, dipeptidyl-peptidase-4 inhibitors; MMSE, Mini-Mental State Examination; GLDs, glucose-lowering drugs apart from insulin; SMD, standardized mean differences; Comparisons per baseline incident exposure assignment – exposure assessed at one-year period prior to dementia diagnosis in subjects without history of exposure before the one-year period; p-values refer to the exposure “Yes” vs exposure “No” comparisons; Age is described as mean (SD); Charlson comorbidity index, Diabetes duration and MMSE are described as median (IQR); all other variables are described as n (%); SMDs were calculated for the matching variables; All cohorts matched using 1:4 ratio; “Total eligible” expresses the number of eligible subjects for propensity-score matching from the original cohort, with % retained after PS matching. [file 13195_2021_934_MOESM3_ESM.docx]

Supplementary table 1. Balance in baseline characteristics among incident users versus non-users of antidiabetic medications – propensity-score matched cohorts

|  | | Metformin Yes  (n=101) | Metformin No  (277) | p | SMD | Insulin Yes  (66) | Insulin No  (263) | p | SMD | Sulfonylurea Yes  (37) | Sulfonylurea No  (147) | p | SMD |
| --- | --- | --- | --- | --- | --- | --- | --- | --- | --- | --- | --- | --- | --- |
| Age | | 78.6 (6.3) | 78.9 (6.8) | 0.65 | 0.09 | 80.4 (6.0) | 80.6 (6.2) | 0.76 | -0.05 | 77.9 (6.6) | 77.9 (8.3) | 0.99 | 0.01 |
| Female | | 58 (57.4%) | 149 (53.8%) | 0.53 |  | 33 (50.0%) | 139 (52.9%) | 0.68 |  | 19 (51.4%) | 70 (47.6%) | 0.69 |  |
| Living alone | | 39 (38.6%) | 116 (41.9%) | 0.25 | 0.05 | 24 (36.4%) | 91 (34.6%) | 0.90 | -0.02 | 15 (40.5%) | 61 (41.5%) | 0.85 |  |
| Institutionalized | | 0 (0.0%) | 6 (2.2%) |  |  | 2 (3.0%) | 6 (2.3%) |  |  | 1 (2.7%) | 2 (1.4%) |  |  |
| Baseline MMSE | | 23 (5) | 22 (6) | 0.029 |  | 23 (6.3) | 22 (6) | 0.96 |  | 22 (5) | 23 (6) | 0.80 |  |
| Alzheimer’s disease | | 49 (48.5%) | 151 (54.5%) | 0.30 |  | 37 (56.1%) | 29 (43.9%) | 0.89 |  | 21 (56.8%) | 90 (61.2%) | 0.62 |  |
| Mixed dementia | | 52 (51.5%) | 126 (45.5%) |  |  | 150 (57.0%) | 113 (43.0%) |  |  | 16 (43.2%) | 57 (38.8% |  |  |
| Diabetes duration | | 0.8 (5.4) | 3.5 (5.5) | <0.001 | -0.05 | 5.8 (5.5) | 5.8 (4.2) | 0.84 | 0.04 | 2.8 (5.6) | 3.0 (5.1) | 0.88 | -0.00 |
| Charlson index | | 1 (2) | 2 (2) | 0.07 | -0.02 | 2 (2) | 2 (2) | 0.82 | -0.01 | 1 (2) | 2 (2) | 0.31 |  |
| Renal disease | | 1 (1.0%) | 4 (1.4%) | 1.00 | 0.00 | 6 (9.1%) | 9 (3.4%) | 0.05 |  | 1 (2.7%) | 5 (3.4%) | 1.00 |  |
| Antihypertensives | | 81 (80.2%) | 202 (72.9%) | 0.15 |  | 51 (77.3%) | 211 (80.2%) | 0.59 |  | 29 (78.4%) | 108 (73.5%) | 0.54 |  |
| Statins | | 66 (65.3%) | 172 (62.1%) | 0.56 |  | 45 (68.2%) | 180 (68.4%) | 0.97 |  | 24 (64.9%) | 93 (63.3%) | 0.86 |  |
| Antithrombotics | | 64 (63.4%) | 183 (66.1%) | 0.63 |  | 44 (66.7%) | 191 (72.6%) | 0.34 |  | 23 (62.2%) | 96 (65.3%) | 0.72 |  |
| Antipsychotics | | 3 (3.0%) | 4 (1.4%) | 0.39 | 0.08 | 2 (3.0%) | 10 (3.8%) | 0.77 |  | 1 (2.7%) | 3 (2.0%) | 0.81 |  |
| Antidepressants | | 30 (29.7%) | 84 (30.3%) | 0.91 | 0.06 | 10 (15.2%) | 91 (34.6%) | 0.002 |  | 8 (21.6%) | 39 (26.5%) | 0.54 |  |
| ChEI | | 24 (23.8%) | 59 (21.3%) | 0.61 | 0.09 | 13 (19.7%) | 51 (19.4%) | 0.96 |  | 11 (29.7%) | 42 (28.6%) | 0.89 | 0.01 |
| Other GLDs | | 22 (21.8%) | 81 (29.2%) | 0.15 | -0.01 | 61 (92.4%) | 238 (90.5%) | 0.63 | 0.07 | 24 (64.9%) | 92 (62.6%) | 0.80 |  |
| Insulin | | 14 (13.9%) | 63 (22.7%) | 0.06 |  |  |  |  |  | 6 (16.2%) | 30 (20.4%) | 0.57 |  |
| Income | Low | 35 (34.7%) | 96 (34.7%) | 1.00 | 0.01 | 28 (42.4%) | 96 (36.5%) | 0.10 |  | 12 (32.4%) | 46 (31.3%) | 0.34 |  |
|  | Middle | 32 (31.7%) | 87 (31.4%) |  |  | 22 (33.3%) | 67 (25.5%) |  |  | 15 (40.5%) | 44 (29.9%) |  |  |
|  | High | 34 (33.7%) | 94 (33.9%) |  |  | 16 (24.2%) | 100 (38.0%) |  |  | 10 (27.0%) | 57 (38.8%) |  |  |
| Total eligible | | 114 (88.6%) | 532 (52.2%) |  |  | 66 (100%) | 1,228 (21.4%) |  |  | 37 (100%) | 1,209 (12.3%) |  |  |

ChEI, cholinesterase inhibitors; DPP-4i, dipeptidyl-peptidase-4 inhibitors; MMSE, Mini-Mental State Examination; GLDs, glucose-lowering drugs apart from insulin including glucagon-like peptide-1 analogues; SMD, standardized mean differences; Comparisons per baseline incident exposure assignment – exposure assessed at one-year period prior to dementia diagnosis in subjects without history of exposure before the one-year period; p-values refer to the exposure “Yes” vs exposure “No” comparisons; Age is described as mean (SD); Charlson comorbidity index, Diabetes duration and MMSE are described as median (IQR); all other variables are described as n (%); SMDs were calculated for the matching variables; All cohorts matched using 1:4 ratio; “Total eligible” expresses the number of eligible subjects for propensity-score matching from the original cohort, with % retained after PS matching;
