# Supplementary material for: The association of antidiabetic medications and Mini-Mental State Examination scores in patients with diabetes and dementia
Source: Alzheimers Res Ther. 2021 Dec 2;13:197. doi: 10.1186/s13195-021-00934-0 (PMC8641148; doi:10.1186/s13195-021-00934-0)
Supplement: Supplementary file 4 — Additional file 4: Supplementary Table 2. Dropout rate and number of follow-ups in observed and imputed data by users of antidiabetic medications. DPP-4i, dipeptidyl-peptidase-4 inhibitors; TZD, thiazolidinediones; Dropout was counted when the patient had neither died nor did the study end occur in the year following the last observed MMSE; Number of follow-ups are expressed in number of follow-ups in total per drug user, and percentage from baseline number of users; Imputed follow-ups were based on the slope of change between observed MMSE measurements and time from baseline. [file 13195_2021_934_MOESM4_ESM.docx]

Supplementary table 2. Dropout rate and number of follow-ups in observed and imputed data by users of antidiabetic medications

| Baseline users, n  PS-matched cohort | | | 1^st^ follow-up  n (% of baseline) | 2^nd^ | 3^rd^ | 4^th^ | 5^th^ | 6^th^ | 7^th^ | >7 | Total dropout,  n (%) |
| --- | --- | --- | --- | --- | --- | --- | --- | --- | --- | --- | --- |
| Prevalent | Metformin (514 users) | Observed | 514 (100%) | 163 (31.7%) | 60 (11.7%) | 26 (5.1%) | 8 (1.6%) | 5 (1.0%) | 2 (0.4%) | n/a | 376 (73.2%) |
|  |  | Imputed | 514 (100%) | 314 (61.1%) | 196 (38.1%) | 119 (23.2%) | 76 (14.8%) | 61 (11.9%) | 40 (7.8%) | 26 (5.1%) |  |
|  | Insulin  (543 users) | Observed | 543 (100%) | 179 (33.0%) | 64 (11.8%) | 32 (5.9%) | 12 (2.2%) | 5 (0.9%) | 2 (0.4%) | n/a | 413 (76.1%) |
|  |  | Imputed | 543 (100%) | 312 (57.5%) | 209 (38.5%) | 123 (22.7%) | 87 (16.0%) | 69 (12.7%) | 45 (8.3%) | 32 (5.9%) |  |
|  | Sulfonylurea (640 users) | Observed | 640 (100%) | 188 (29.4%) | 68 (10.6%) | 31 (4.8%) | 15 (2.3%) | 8 (1.3%) | 2 (0.3%) | n/a | 489 (76.4%) |
|  |  | Imputed | 640 (100%) | 359 (56.1%) | 227 (35.5%) | 145 (22.7%) | 94 (14.7%) | 68 (10.6%) | 45 (7.0%) | 32 (5.0%) |  |
|  | TZD  (67 users) | Observed | 67 (100%) | 28 (41.8%) | 10 (14.9%) | 6 (9.0%) | 2 (3.0%) | 1 (1.5%) | 1 (1.5%) | n/a | 51 (76.1%) |
|  |  | Imputed | 67 (100%) | 43 (64.2%) | 32 (47.8%) | 19 (28.4%) | 13 (19.4%) | 10 (14.9%) | 8 (11.9%) | 2 (3.0%) |  |
|  | DPP-4i  (103 users) | Observed | 103 (100%) | 28 (27.2%) | 3 (3.0%) | 1 (1.0%) | n/a | n/a | n/a | n/a | 63 (61.2%) |
|  |  | Imputed | 103 (100%) | 52 (50.5%) | 35 (34.0%) | 17 (16.5%) | 10 (9.7%) | 7 (6.8%) | 4 (3.9%) | 1 (1.0%) |  |
| Incident | Metformin  (101 users) | Observed | 101 (100%) | 34 (33.7%) | 15 (14.9%) | 5 (5.0%) | 3 (3.0%) | 1 (1.0%) | n/a | n/a | 68 (67.3%) |
|  |  | Imputed | 101 (100%) | 69 (68.3%) | 44 (43.6%) | 27 (26.7%) | 20 (19.8%) | 14 (13.9%) | 7 (6.9%) | 7 (6.9%) |  |
|  | Insulin  (66 users) | Observed | 66 (100%) | 20 (30.3%) | 5 (7.6%) | 2 (3.0%) | 2 (3.0%) | n/a | n/a | n/a | 55 (83.3%) |
|  |  | Imputed | 66 (100%) | 35 (53.0%) | 23 (34.8%) | 15 (22.7%) | 8 (12.1%) | 7 (10.6%) | 4 (6.1%) | 4 (6.1%) |  |
|  | Sulfonylurea  (37 users) | Observed | 37 (100%) | 13 (35.1%) | 6 (16.2%) | 4 (10.8%) | 1 (2.7%) | n/a | n/a | n/a | 32 (86.5%) |
|  |  | Imputed | 37 (100%) | 25 (67.6%) | 16 (43.2%) | 12 (32.4%) | 8 (21.6%) | 5 (13.5%) | 4 (10.8%) | 3 (8.1%) |  |
|  | Whole cohort (1,873 subjects) | Observed | 1,873 (100%) | 611 (32.6%) | 216 (11.5%) | 95 (5.1%) | 39 (2.1%) | 20 (1.1%) | 5 (0.3%) | n/a | 1,382 (73.8%) |
|  |  | Imputed | 1,873 (100%) | 725 (38.7%) | 448 (23.9%) | 287 (15.3%) | 207 (11.1%) | 136 (7.3%) | 90 (4.8%) | 62 (3.3%) |  |

DPP-4i, dipeptidyl-peptidase-4 inhibitors; TZD, thiazolidinediones; Dropout was counted when the patient had neither died nor did the study end occur in the year following the last observed MMSE; Number of follow-ups are expressed in number of follow-ups in total per drug user, and percentage from baseline number of users; Imputed follow-ups were based on the slope of change between observed MMSE measurements and time from baseline
