# Supplementary material for: The association of antidiabetic medications and Mini-Mental State Examination scores in patients with diabetes and dementia
Source: Alzheimers Res Ther. 2021 Dec 2;13:197. doi: 10.1186/s13195-021-00934-0 (PMC8641148; doi:10.1186/s13195-021-00934-0)
Supplement: Supplementary file 5 — Additional file 5: Supplementary Table 3. Baseline differences in the propensity-score matched comparison pairs of the non-metformin antidiabetic drug users. AD, Alzheimer’s disease; ChEI, cholinesterase inhibitors; DPP-4i, dipeptidyl-peptidase-4 inhibitors; MixDem, mixed-pathology dementia; MMSE, Mini-Mental State Examination; GLDs, glucose-lowering drugs apart from insulin; SMD, standardized mean differences; SMDs were calculated for the matching variables; Matching was restricted by number of available users; Insulin vs sulfonylurea analysis was matched using 1:1 ratio, otherwise 1:4 ratio was used; “Total eligible” expresses the number of eligible subjects for propensity-score matching from the original cohort, with % retained after PS matching. [file 13195_2021_934_MOESM5_ESM.docx]

Supplementary table 3. Baseline differences in the propensity-score matched comparison pairs of the non-metformin antidiabetic drug users

|  | | Insulin  (n=259) | Sulfonylurea  (259) | p | SMD | Insulin  (138) | TZD  (35) | p | SMD | Insulin  (199) | DPP-4i  (51) | p | SMD |
| --- | --- | --- | --- | --- | --- | --- | --- | --- | --- | --- | --- | --- | --- |
| Age | | 78.6 (6.0) | 78.8 (6.3) | 0.76 | -0.02 | 75.8 (7.1) | 75.8 (6.5) | 0.97 | 0.00 | 77.4 (7.4) | 77.7 (5.9) | 0.81 | 0.05 |
| Female | | 134 (51.7%) | 140 (54.1%) | 0.60 | 0.05 | 75 (54.3%) | 18 (51.4%) | 0.76 |  | 86 (43.2%) | 22 (43.1%) | 0.99 |  |
| Living alone | | 99 (38.2%) | 102 (39.4%) | 0.26 | 0.05 | 43 (31.2%) | 7 (20.0%) | 0.43 |  | 63 (31.7%) | 16 (31.4%) | 0.92 | -0.03 |
| Institutionalized | | 5 (1.9%) | 1 (0.4%) |  |  | 3 (2.2%) | 1 (2.9%) |  |  | 6 (3.0%) | 1 (2.0%) |  |  |
| Baseline MMSE | | 23 (5) | 22 (6) | 0.47 |  | 23 (7) | 22 (5) | 0.87 |  | 22 (6) | 23 (6) | 0.97 |  |
| AD | | 148 (57.1%) | 156 (60.2%) | 0.48 | 0.06 | 74 (53.6%) | 26 (74.3%) | 0.03 |  | 102 (51.3%) | 29 (56.9%) | 0.47 |  |
| MixDem | | 111 (42.9%) | 103 (39.8%) |  |  | 64 (46.4%) | 9 (25.7%) |  |  | 97 (48.7%) | 22 (43.1%) |  |  |
| Diabetes duration | | 7.7 (5.1) | 7.9 (4.6) | 0.58 | -0.03 | 7.7 (4.5) | 6.8 (3.5) | 0.68 | -0.01 | 8.4 (5.0) | 8.1 (3.5) | 0.92 | -0.02 |
| Charlson index | | 1 (1) | 1 (1) | 0.23 | 0.03 | 2 (2) | 1 (1) | 0.06 |  | 2 (2) | 1 (2) | 0.47 | -0.01 |
| Renal disease | | 12 (4.6%) | 9 (3.5%) | 0.50 | 0.05 | 5 (3.6%) | 1 (2.9%) | 0.83 |  | 11 (5.5%) | 3 (5.9%) | 1.00 |  |
| Antihypertensives | | 203 (78.4%) | 208 (80.3%) | 0.59 | -0.05 | 105 (76.1%) | 27 (77.1%) | 0.90 |  | 163 (81.9%) | 40 (78.4%) | 0.57 |  |
| Statins | | 190 (73.4%) | 192 (74.1%) | 0.84 | -0.02 | 110 (79.7%) | 27 (77.1%) | 0.74 |  | 151 (75.9%) | 46 (90.2%) | 0.03 |  |
| Antithrombotics | | 179 (69.1%) | 177 (68.3%) | 0.85 | 0.02 | 105 (76.1%) | 21 (60.0%) | 0.06 |  | 152 (76.4%) | 40 (78.4%) | 0.76 |  |
| Antipsychotics | | 7 (2.7%) | 7 (2.7%) | 1.00 | 0.00 | 5 (3.6%) | 2 (5.7%) | 0.58 |  | 5 (2.5%) | 4 (7.8%) | 0.07 |  |
| Antidepressants | | 78 (30.1%) | 82 (31.7%) | 0.70 | -0.03 | 46 (33.3%) | 12 (34.3%) | 0.92 |  | 58 (29.1%) | 12 (23.5%) | 0.43 |  |
| ChEI | | 63 (24.3%) | 65 (25.1%) | 0.84 | -0.02 | 41 (29.7%) | 10 (28.6%) | 0.90 | -0.03 | 46 (23.1%) | 12 (23.5%) | 0.95 | 0.02 |
| Other GLDs | | 196 (75.7%) | 191 (73.7%) | 0.61 | 0.04 | 114 (82.6%) | 33 (94.3%) | 0.08 |  | 171 (85.9%) | 49 (96.1%) | 0.05 |  |
| Income | Low | 88 (34.0%) | 92 (35.5%) | 0.88 | 0.01 | 50 (36.2%) | 13 (37.1%) | 0.23 |  | 70 (35.2%) | 15 (29.4%) | 0.59 |  |
|  | Middle | 80 (30.9%) | 75 (29.0%) |  |  | 38 (27.5%) | 14 (40.0%) |  |  | 58 (29.1%) | 14 (27.5%) |  |  |
|  | High | 91 (35.1%) | 92 (35.5%) |  |  | 50 (36.2%) | 8 (22.9%) |  |  | 71 (35.7%) | 22 (43.1%) |  |  |
| Total eligible | | 359 (72.1%) | 378 (68.5%) |  |  | 612 (22.5%) | 35 (100%) |  |  | 593 (33.6%) | 51 (100%) |  |  |
|  | | Sulfonylurea  (111) | TZD  (31) | p | SMD | Sulfonylurea  (141) | DPP-4i  (38) | p | SMD | DPP-4i  (45) | TZD (45) | p | SMD |
| Age | | 76.2 (5.5) | 75.4 (5.9) | 0.48 | -0.01 | 76.4 (6.4) | 76.1 (5.8) | 0.84 | 0.01 | 77.8 (5.3) | 77.6 (5.5) | 0.80 | 0.05 |
| Female | | 43 (38.7%) | 13 (41.9%) | 0.75 |  | 72 (51.1%) | 18 (47.4%) | 0.69 |  | 19 (42.2%) | 26 (57.8%) | 0.52 |  |
| Living alone | | 35 (31.5%) | 7 (22.6%) | 0.45 |  | 47 (33.3%) | 10 (26.3%) | 0.31 |  | 14 (31.1%) | 13 (28.9%) | 1.00 | -0.05 |
| Institutionalized | | 2 (1.8%) | 0 (0.0%) |  |  | 5 (3.5%) | 0 (0.0%) |  |  | 0 (0.0%) | 0 (0.0%) |  |  |
| Baseline MMSE | | 22 (5) | 24 (6) | 0.22 |  | 23 (5) | 22 (8.3) | 0.57 |  | 24 (5) | 22 (5.5) | 0.19 |  |
| AD | | 60 (54.1%) | 23 (74.2%) | 0.04 |  | 73 (51.8%) | 23 (60.5%) | 0.34 |  | 21 (46.7%) | 28 (62.2%) | 0.14 |  |
| MixDem | | 51 (45.9%) | 8 (25.8%) |  |  | 68 (48.2%) | 15 (39.5%) |  |  | 24 (53.3%) | 17 (37.8%) |  |  |
| Diabetes duration | | 7.9 (4.5) | 8.3 (5.2) | 0.49 | 0.01 | 8.8 (5.0) | 8.5 (5.5) | 0.83 | 0.02 | 8.7 (3.7) | 8.0 (4.4) | 0.51 | 0.03 |
| Charlson index | | 2 (1) | 2 (2) | 0.98 |  | 2 (2) | 2 (2) | 0.80 |  | 2 (1.5) | 1 (1.5) | 0.45 | 0.06 |
| Renal disease | | 4 (3.6%) | 1 (3.2%) | 1.00 |  | 3 (2.1%) | 3 (7.9%) | 0.08 |  | 5 (11.1%) | 2 (4.4%) | 0.24 |  |
| Antihypertensives | | 92 (82.9%) | 27 (87.1%) | 0.57 |  | 117 (83.0%) | 31 (81.6%) | 0.84 |  | 35 (77.8%) | 36 (80.0%) | 0.80 |  |
| Statins | | 85 (76.6%) | 21 (67.7%) | 0.32 |  | 106 (75.2%) | 37 (97.4%) | 0.002 |  | 41 (91.1%) | 32 (71.1%) | 0.02 |  |
| Antithrombotics | | 76 (68.5%) | 18 (58.1%) | 0.28 |  | 105 (74.5%) | 31 (81.6%) | 0.36 |  | 38 (84.4%) | 29 (64.4%) | 0.03 |  |
| Antipsychotics | | 3 (2.7%) | 1 (3.2%) | 1.00 |  | 4 (2.8%) | 2 (5.3%) | 0.46 |  | 2 (4.4%) | 2 (4.4%) | 1.00 |  |
| Antidepressants | | 35 (31.5%) | 10 (32.3%) | 0.94 |  | 49 (34.8%) | 7 (18.4%) | 0.05 |  | 11 (24.4%) | 12 (26.7%) | 0.81 |  |
| ChEI | | 25 (22.5%) | 8 (25.8%) | 0.70 | 0.10 | 29 (20.6%) | 7 (18.4%) | 0.77 | -0.04 | 10 (22.2%) | 8 (17.8%) | 0.60 | 0.12 |
| Other GLDs | | 83 (74.8%) | 29 (93.5%) | 0.02 |  | 117 (83.0%) | 33 (86.8%) | 0.57 |  | 43 (95.6%) | 44 (97.8%) | 0.56 |  |
| Insulin | | 50 (45.0%) | 10 (32.3%) | 0.20 |  | 71 (50.4%) | 20 (52.6%) | 0.80 |  | 21 (46.7%) | 20 (44.4%) | 0.83 |  |
| Income | Low | 41 (36.9%) | 11 (35.5%) | 0.95 |  | 45 (31.9%) | 12 (31.6%) | 0.97 |  | 8 (17.8%) | 11 (24.4%) | 0.70 |  |
|  | Middle | 29 (26.1%) | 9 (29.0%) |  |  | 38 (27.0%) | 11 (28.9%) |  |  | 20 (44.4%) | 17 (37.8%) |  |  |
|  | High | 11 (35.5%) | 11 (35.5%) |  |  | 58 (41.1%) | 15 (39.5%) |  |  | 17 (37.8%) | 17 (37.8%) |  |  |
| Total eligible | | 627 (17.7%) | 31 (100%) |  |  | 599 (23.5%) | 38 (100%) |  |  | 89 (50.6%) | 54 (83.3%) |  |  |

AD, Alzheimer’s disease; ChEI, cholinesterase inhibitors; DPP-4i, dipeptidyl-peptidase-4 inhibitors; MixDem, mixed-pathology dementia; MMSE, Mini-Mental State Examination; GLDs, glucose-lowering drugs apart from insulin including glucagon-like peptide-1 analogues; SMD, standardized mean differences; SMDs were calculated for the matching variables; Matching was restricted by number of available users; Insulin vs sulfonylurea analysis was matched using 1:1 ratio, otherwise 1:4 ratio was used; “Total eligible” expresses the number of eligible subjects for propensity-score matching from the original cohort, with % retained after PS matching
